# Tuberculosis alters pancreatic enzymes in the absence of pancreatitis

**DOI:** 10.4102/ajlm.v3i1.129

**Published:** 2014-10-16

**Authors:** Modisa S. Motswaledi, Rosinah Sekgwama, Ishmael Kasvosve

**Affiliations:** 1 Faculty of Health Sciences, Department of Medical Laboratory Sciences, University of Botswana, Botswana; 2 Jwaneng Mine Hospital, Jwaneng, Botswana

## Abstract

**Background:**

Lipases and phospholipases are thought to contribute to the pathogenesis of *Mycobacterium tuberculosis* (MTB) infection. Genes coding for lipases, phospholipases and amylase are present in MTB, enabling the bacteria to produce these enzymes.

**Objective:**

To compare serum lipase and amylase activity levels in patients with tuberculosis (TB) against those of healthy controls.

**Methods:**

Serum lipase and amylase activity levels were measured in 99 patients and 143 healthy controls using the Vitros 250 Chemistry analyser. Reference ranges for serum lipase and amylase were 23–300 U/L and 30–110 U/L, respectively.

**Results:**

Lipase was higher in patients with MTB than in controls (81.5 IU/L versus 66.5 IU/L, *p* = 0.006). Similarly, amylase was higher in the MTB patient group (76 IU/L versus 60 IU/L, *p* < 0.001). The Pearson correlation coefficient for lipase versus amylase (*R*) was higher in the controls (*R* = 0.351, *p* < 0.0001) compared with MTB patients (*R* = 0.217, *p* = 0.035). Amongst MTB patients, lipase activity correlated positively with erythrocyte sedimentation rate (ESR) (*R* = 0.263, *p* = 0.013), but not with haemoglobin concentration or treatment duration. A weak inverse correlation was noted between ESR and treatment duration (*R* = -0.222, *p* = 0.028).

**Conclusion:**

Pancreatic enzyme levels differ between MTB patients and normal controls; however, this difference still lies within the normal range. The concomitant increase of lipase with ESR, an inflammatory marker, could conceivably suggest a causal relationship. Further research is necessary to characterise MTB-derived enzymes for diagnostic and therapeutic utility.

## Introduction

Serum lipase and amylase activity levels are used traditionally in the diagnosis of pancreatitis. However, in recent years, many investigators have demonstrated the production of pancreatic enzymes in infectious diseases that do not necessarily involve the pancreas.^1,2^ Enzymes with lipolytic activity have been implicated as the cause of cytopathic effects on macrophages through lysis of host cell membrane lipids^2,3,4,5^ and have been suggested as potential biomarkers for active tuberculosis (TB) infection.^6^ The identification of genes in *Mycobacterium tuberculosis* (MTB) coding for these enzymes has further corroborated the case for their involvement in the pathogenesis of TB.^2,7,8,9,10,11,12^

The purpose of this research was therefore to investigate whether serum activity levels of lipase are elevated in patients with MTB infection. Serum amylase was included in order to exclude lipase elevations resulting from pancreatitis, when activity of both enzymes would be expected to be high. It was hypothesised that if lipase enzymatic activity is altered during TB infection, such activity should have a positive correlation with erythrocyte sedimentation rate (ESR), a marker of active inflammation, but should diminish with treatment duration. On the other hand, the resolution of infection after treatment was expected to result in an improvement of the haemoglobin (HB) level, which is thus a negative correlation.

Lipase production has been demonstrated in different microorganisms, including *Staphylococcus aureus*.^13^ A lipase that inhibits the phagocytic activity of alveolar macrophages has been described in *Pseudomonas cetacean*,^14^ whilst yet another lipolytic enzyme has been shown to be important for the penetration of endocervical epithelial cells in *Neisseria gonorrhoeae* infection.^15^ Similarly, *Yersinia enterocolitica’*s phospholipase A has been demonstrated to contribute to bacterial pathogenesis in mouse models.^16^ MTB is known to possess genes coding for several lipases, including phospholipases A2, C and D.^3,4,6,9^ These reports suggest a new role of pancreatic enzymes in non-pancreatic, infectious disorders.

Phospholipases are particularly important in bacterial interactions with host cell membranes which provide the substrate for such enzymes, leading to cell lysis.^3^ Cell lysis as a result of phospholipase activity could be important for explaining some forms of tissue necrosis, anaemia and low blood cell counts observed in many infectious diseases, including TB.^5,17^ The accumulation of membrane lipid components in the MTB caseum such as triacylglycerides, cholesterols and lactosylceramides provides further evidence that MTB possesses the enzymes necessary for degrading membrane lipids.^12^ In this connection, Bakala N’goma et al. demonstrated that phospholipase C from MTB induced cytopathic effects on murine macrophages.^3^ Lee et al. described hydrolysis of cell membrane lipid bi-layers that was triggered by excessive numbers of mycobacteria per cell and postulated that this is the mycobacterium’s escape mechanism from cells that can no longer support the bacterial load.^5^ In fact, Deb et al. had demonstrated previously that mycobacterial LipY, also referred to as Rv3097c, was activated under hypoxic conditions and was responsible for the degradation of the caseum-stored triacylglycerides in the face of limited nutrients.^10^

MTB synthesises as well as acquires lipids from its host cell membranes. These lipids are then utilised as energy sources during the reactivation phase by employing lipolytic enzymes.^2,6^ Various lipolytic enzymes have been ascribed to MTB, including those that hydrolyse monoacylglycerides, triacylglycerides, lipids and phospholipids.^3,4^ Brust et al. demonstrated that patients with active MTB infection produce immunoglobulin G antibodies against certain mycobacterial lipases (LipY, Rv0183, Rv1984c and Rv3452).^6^ However, such antibodies were not detected in Bacille Calmette Guerin (BCG)-vaccinated individuals, suggesting that they were elicited by active live mycobacteria. Côtes and colleagues identified and characterised a monoacylglycerol lipase that was exported to the extracellular medium, indicating the possibility of mycobacterial enzymes acting at sites remote from the focus of infection.^9^ The findings of Brust et al. also documented the presence of lipolytic enzymes in MTB and reported that Rev3452 exhibited phospholipase A2 activity with the ability to lyse macrophages.^6^

MTB therefore possesses the ability to hydrolyse phospholipids and lysophospholipids at virtually any position using enzymes such as LipY (a triacylglycerol hydrolase),^18^ Rv3452 (a phospholipase A2),^4^ Rv1984c (acting on medium-length chain carboxylic esters and monoacylglycerol),^4^ Rv0183 (diacyl and triacylglycerols),^2^ Rv1399c (soluble triacylglycerol and vinyl esters).^7^

Dhouib et al., working with a recombinant protein from *Mycobacterium smegmatis* exhibiting lipase activity (MSMEG_0220), showed that the protein had extensive amino acid sequence identity with the mycobacterial lipase Rv0183.^19^ A mutation of this gene impaired the ability of the bacterium to utilise mono-olein for growth and increased its susceptibility to rifampicin, but imparted more resistance against isoniazid. An understanding of the role of lipases in mycobacterial infections may therefore provide insight into their interactions with chemotherapeutic agents.

Amylase, on the other hand, is an enzyme almost exclusively devoted to the digestive system. However, the product of the gene Rv1326c from MTB exhibits both the glycogen branching and α-amylase activities.^11^ A study by Villena et al., involving 40 neoplastic lesions and 26 benign conditions, found that TB was the most frequent non-neoplastic cause of a high serum amylase. Moreover, high levels of serum amylase correlated with worse survival.^1^ The function of amylase in mycobacterial metabolism remains to be unravelled.

## Research method and design

Patients were recruited at various TB treatment facilities by explaining the purpose and procedure of the study. Written, informed consent (in both Setswana and English) was obtained from each consenting individual 18 years and older. Participants were informed of their right to withdraw from the study at any time without fear of victimisation or compromised care. Parental permission was obtained prior to obtaining samples from persons under the age of 21 years. Ninety-nine patients of all ages with MTB, who were receiving standard anti-TB treatment at 12 clinics in Botswana, were studied. Testing was done at Jwaneng Mine Hospital.

All patients in the study were diagnosed with TB and were at various stages of treatment, including those who were commencing treatment, those who had been on treatment for some time and those being managed for relapses of pulmonary or extrapulmonary TB. The patients selected for this study were neither being managed for renal insufficiency nor was there any clinical evidence of inflammatory conditions other than TB. Written informed consent was obtained from the patients. For children aged less than 18 years, permission was obtained from their parents and/or guardians. Demographic data and other data needed for the research were retrieved from patients’ medical records. Control samples were obtained from the National Blood Transfusion Service. These were selected on the basis of having undergone stringent health screening and been found to be free of any signs of acute or chronic diseases to ensure the safety of their blood for transfusion. All samples with an enzyme activity level four times the upper limit of normal or more, for either of the enzymes under study (lipase or amylase), were excluded. Levels in this range defined pancreatitis. None of the patients included in the computation had amylase or lipase activity levels elevated sufficiently to suggest pancreatitis defined in this manner.

### Ethical considerations

Permission to carry out research was obtained from the University of Botswana’s Institutional Review Board, the Botswana Ministry of Health (Permit no. PPME13/18/1 US V [184]), District Health Management Teams (AH 10/7 I [79] plus Gaborone DHMT letter dated 17/01/2012) and the Jwaneng District Health Management Team (JDHMT PF 58). Demographic data were entered in a password-protected computer and no details of names or personal identifiers were used. Access to data was limited to the researchers only.

Venous blood was collected following standard safety and operating procedures. For the serum lipase and amylase assays, 3 mL – 5 mL of blood was collected in plain tubes. For the ESR and full blood count assays, 3 mL – 5 mL of blood was collected in tri-potassium ethylene diamine tetra acetic acid (K_3_EDTA) tubes. For transportation, samples were stored in a cooler box with ice packs and processed on the day of collection. Blood in plain tubes was centrifuged at 3500 rpm for 5 minutes and the serum was transferred into micro vials, stored at 2–8 ^°^C in a refrigerator and processed the following day in accordance with the manufacturer’s (Vitros Chemistry Products, Ortho Clinical Diagnostics, Rochester, NY) recommendation that lipase and amylase activity levels are stable for 3 weeks at refrigerator temperatures.^20,21^

Serum lipase and amylase activity levels were measured using the Vitros 250 Chemistry analyser (Ortho Clinical Diagnostics, Rochester, NY) using reagents, control sera and test protocols supplied by the manufacturer. Only lipase and amylase were measured in the control group. Haemoglobin concentration was measured in samples from MTB patients, using the Sysmex® XT2000i (Sysmex® Asia Pacific, Woodlands, Singapore) and ESR was determined using the Westergren method.^22^

In this study, pancreatitis was defined as an amylase activity level at least four times the upper limit of normal (≥ 440 IU/L) or lipase at least five times the upper limit of normal (≥ 1500 IU/L).^23^ Statistical analysis was performed using IBM SPSS version 20 statistical software (IBM Corporation, Somers, NY). The independent samples Mann-Whitney U test was used to test for differences in lipase and amylase activity level between patients with MTB and controls and Pearson correlation analysis was used to determine relationships between lipase, amylase, ESR, haemoglobin concentrations and treatment duration in the patient and control groups. For purposes of calculations, ESR values above 150 mm/hr and amylase values below the detection level (< 31 IU/L) were excluded and not used in any of the calculations. Treatment duration data were rounded to the nearest whole month.

## Results

Serum lipase and amylase activity levels were measured in 99 MTB-infected individuals and 143 uninfected controls. The results of one patient with TB were excluded from the analysis since their lipase and amylase levels were consistent with pancreatitis as defined above.^23^ Lipase levels were higher (81.5 IU/L) in MTB patients than in the controls (66.5 IU/L), *p* = 0.006, as were amylase levels (patients 76 IU/L versus controls 60 IU/L, *p* < 0.001). The enzyme level data distribution was non-Gaussian, hence the choice for using the median rather than the conventional mean. The results are summarised in Table 1.

**TABLE 1:** Comparison of lipase and amylase activity levels in patients with tuberculosis versus normal controls.

Variables	MTB patients *n* = 98	Controls (normal) *n* = 143	*p*-value
	Median (IQR)	Median (IQR)
Lipase activity, IU/L	81.5 (57–122)	66.5 (45–110)	0.006
Amylase activity, IU/L	76 (61–93)	60 (44–71)	< 0.001
Pearson correlation (R) for lipase versus amylase	0.217*	0.351**	-
HB versus ESR	-0.420	-	< 0.001
ESR versus treatment duration	-0.222	-	0.028

Note: Median values were higher for both enzymes in the patient group. Results for one MTB patient were excluded as lipase and amylase levels were consistent with pancreatitis. IQR, Inter quartile range; MTB, *Mycobacterium tuberculosis*; HB, haemoglobin; ESR, erythrocyte sedimentation rate.

* *p*-value, 0.035;

** *p*-value < 0.0001

In the controls, lipase and amylase levels correlated significantly (Pearson correlation coefficient *R* = 0.351, *p* < 0.0001). This relationship, although still present in the MTB patient group, was weaker (*R* = 0.217, *p* = 0.035). As expected, ESR correlated negatively with both HB (*R* = -0.420, *p* < 0.001) and treatment duration (*R* = -0.222, *p* = 0.028). The mean values (standard deviation) for other parameters were: ESR = 66 (40) mm/hr; HB = 11.8 (2.5) g/dL; and treatment duration = 3.1 (2) months.

Lipase levels in the MTB patient group correlated positively with ESR, a surrogate test for inflammation. Amylase levels in patients did not correlate with any of the parameters measured. The results are summarised in Table 2.

**TABLE 2:** Lipase and amylase Pearson correlation statistics in MTB patients versus other parameters.

Variables	Correlation coefficient (*R*)	*p*-value
**Lipase versus**		
ESR	0.263	0.013
HB	-0.174	0.087
Treatment duration	0.049	0.629
**Amylase versus**		
ESR	0.019	0.856
HB	-0.028	0.786
Treatment duration	-0.058	0.570

MTB, *Mycobacterium tuberculosis*; ESR, erythrocyte sedimentation rate; HB, haemoglobin.

## Discussion

We measured lipase and amylase activity levels in MTB-infected individuals and normal controls. Lipase and amylase activity levels were significantly higher in the MTB patient group. Moreover, lipase correlated with severity of inflammation as estimated by the ESR, leading us to infer that lipase is conceivably causally related to the underlying inflammatory process.

The serum lipase levels in normal controls correlated with those of amylase, suggesting a common pancreatic source. Although the lipase-amylase relationship was weak, it was clearly stronger in the control group than among MTB patients. The weakening of this correlation in infected individuals could suggest production of the enzymes at independent, non-pancreatic sites in MTB where genes are regulated by varying or independent physiological parameters. Mycobacterial lipase Rv1399c, for example, is a hormone-sensitive hydrolase, whilst production of R3097c is only stimulated under hypoxic and nutrient-deficient conditions.^10^ In addition, the bacterial load, which differs from one individual to another, may also influence the degree to which released mycobacterial enzymes affect the total enzyme activity level. This could be complicated further by the presence of antibodies against mycobacterial lipases in infected individuals.^24^

It remains to be shown by further enquiry whether the enzyme elevations in our study represent de novo mycobacterial activity rather than pancreatitis or the effect of anti-TB drugs.^25,26^ In this study, the lipase and amylase levels were far below those that define pancreatitis, thus technically excluding pancreatic inflammation. Moreover, treatment duration with anti-TB drugs did not correlate with lipase or amylase activity levels. It should be noted, however, that anti-TB drugs could either elevate the enzymes if they caused pancreatitis, or diminish MTB-derived enzymes if treatment were successful.

Patients with TB typically present with an elevated ESR and anaemia.^27^ The ESR is affected by both the anaemia and the underlying inflammation.^28^ The anaemia of chronic infection is generally considered to be a result of ferrokinetics as regulated by the balance between hepcidin and ferroportin.^29^ Although trending negative as expected, the relationship between lipase and HB was not significant in this study. Our results suggest that lipase should be investigated as a possible contributor to haemolysis, anaemia and elevated ESR in the light of other reports of phospholytic activity in patients with TB, which is known to undermine the integrity of cell membranes.^3,9,16,30^

### Limitations of the study

The assays used in this study have not been validated for MTB enzymes. However, mycobacterial lipases are diverse, including several lipases with mono-acyl-, di-acyl- and tri-acyl-glycerol hydrolase activity, as well as multiple phospholipases which should, in principle, be able to act on the triglycerides used as a substrate in this assay. The use of months rather than days to measure the treatment duration may have decreased the sensitivity of the study with regard to making distinctions between new entrants and those who had already undergone treatment for some days. We recommend that future studies include collection of baseline (pre-treatment) samples for each patient, with subsequent or serial samples taken for appropriate statistical comparison.

### Recommendations

Further investigations are necessary to explain the unknown role of amylase in tuberculosis. Furthermore, future research must focus on distinguishing mycobacterial enzymes from those of pancreatic origin so as to improve the interpretation of pancreatic enzyme levels.

### Conclusion

Our findings suggest that lipase and amylase activity levels are elevated mildly in patients with TB. The cause of this elevation is not known. Lipase enzyme activity levels appeared to increase with increasing ESR, a marker of inflammation, which in turn diminished with duration of treatment, which suggests a possible effect of mycobacteria on lipase levels. Although amylase enzyme levels were elevated in patients, there was no correlation with any of the parameters measured and no explanation could be found for this phenomenon, thus the role of amylase in TB remains enigmatic.

Our method, however, did not distinguish between the various enzymes with lipolytic activity. Therefore, development of specific assays for mycobacteria-specific lipases and amylases is recommended and may yield definitive tools for the diagnosis and monitoring of mycobacteria-related diseases.
